# A Community-Based Walk-In Screening of Depression in Taiwan

**DOI:** 10.1155/2014/184018

**Published:** 2014-07-15

**Authors:** Shu-Yu Tai, Tzu-Chiao Ma, Ling-Chun Wang, Yuan-Han Yang

**Affiliations:** ^1^Department of Family Medicine, Kaohsiung Municipal Ta-Tung Hospital, Kaohsiung City 80145, Taiwan; ^2^Department of Family Medicine, Kaohsiung Medical University Hospital, Kaohsiung Medical University, Kaohsiung City 80708, Taiwan; ^3^Mentality Protection Center, Fo Guang Shan Compassion Foundation, Kaohsiung City 80050, Taiwan; ^4^Graduate Institute of Oral Health Sciences, Kaohsiung Medical University, Kaohsiung City 80708, Taiwan; ^5^Department of Neurology, Kaohsiung Medical University Hospital, Kaohsiung Medical University, Kaohsiung City 80708, Taiwan; ^6^Department of Neurology and Master's Program in Neurology, Faculty of Medicine, Kaohsiung Medical University, Kaohsiung City 80708, Taiwan; ^7^Department of Neurology, Kaohsiung Municipal Ta-Tung Hospital, Kaohsiung Medical University, Kaohsiung City 80145, Taiwan

## Abstract

Depression is a crucial public health problem because of its relatively high association with suicidal attempts, prolonged social isolation, poor physical health, and dementia. However, the available data and study on the prevalence of depression in Taiwan were mostly completed within the previous 1 to 2 decades, and these studies were limited to certain areas or populations. Little is known regarding the current status of depression in Taiwan. We used a brief tool, the Center for Epidemiological Studies Depression Scale (CES-D), to screen depression in 4 areas among the general and aged population. The results showed a higher CES-D score in the southern area among general (mean ± SD: 7.8 ± 8.4) or aged participants (mean ± SD: 7.2 ± 8.0) compared with other areas. The ratio of suspected depression patients was 16.4% of all recruited participants and 13.3% of aged participants. These results may provide information for this public health issue.

## 1. Introduction

Depression is a crucial public health problem because of its relatively high prevalence in the general population [1] and its empirically established association with suicide attempts, prolonged social isolation, and poor physical health [2, 3]. Depression also has a profound effect on well-being, daily functioning, and excessive use of health services [4]. Depression in a community may imply the potential effect of well-being in the general population. Moreover, the aged population has been increasing recently. Several observational studies have reported evidence that depression is a critical issue for those working with older adults, particularly for those working with older adults with Alzheimer's disease (AD) [5–7]. Depression affects numerous older adults [8] and has been associated with poor cognitive function [9]. Depression and dementia may be related in several aspects. First, depressive symptoms often occur among patients with dementia. Second, depression may be a reaction to early cognitive deficits. Third, depression can impair cognitive function, leading to a “pseudo-dementia” presentation. Finally, depression may be a risk factor or early symptom of dementia [5, 7] and its treatment has been associated with improved cognitive and functional status of patients with AD [10–12]. Knowledge of the current prevalence of depression, thus, might have crucial clinical and research implications on dementia.

Currently available data and studies on the prevalence of depression in Taiwan were mostly completed within the previous 1 to 2 decades [13, 14]. These studies [15–17] were also conducted sporadically and separately in a localized area of Taiwan with various study designs [18, 19] and psychometrics; therefore, compiling these data to reflect the overall condition of depression in Taiwan is difficult. These published studies did not have sufficient data to reflect the current status of the aged population [20]. Therefore, little is known of the current status of depression in the general and aged population in Taiwan.

The Center for Epidemiological Studies Depression Scale (CES-D), developed by Radloff (1977), is a brief tool used to screen depression, to assess depressive symptoms both clinically [21] and in the community [3, 22]. Since its publication in 1977, the scale has become one of the most frequently used self-report depressive symptom scales and has been shown to have acceptable psychometric properties, including desirable internal consistency, optimal test-retest reliability, high correlations with significant life events, and clinical diagnosis of depression [1, 3, 23]. CES-D is capable of screening mild depression in the general population and has been used extensively in other countries, including Taiwan, after its validation [24]. A person whose CES-D screening results exceed 16 is considered to have depression [3].

The Mentality Protection Center (MPC) is a nonprofit institution that was established under the Fo Guang Shan Compassion Foundation in 2008 to provide medical and charitable services to the general population worldwide through the hundreds of Fo Gunag Shan branches. Collaborating with the Fo Guang Shan branches scattered in urban, suburban, and rural areas in Northern, Central, Southern, and Eastern Taiwan, the MPC launched the dementia, depression, and sleep disorder-screening projects for older adult populations from 2011 to 2013. All the screening results were recruited to the MPC headquarters for statistical analyses.

## 2. Material and Methods

### 2.1. Interviewer Training

All of our interviewers are senior nurses or other medical-related specialists. Before administering the CES-D, they undergo a series of training courses on depression-related and medical related topics and must practice administering the CES-D to the general population by interning with experienced interviewers and physicians. All of the interviewers are MPC volunteers and have completed all walk-in screenings in this project in Taiwan.

### 2.2. Walk-In Screening

The MPC in Taiwan is composed of 59 branches, which are scattered in Northern, Central, Southern, and Eastern Taiwan and distributed in urban, suburban, and rural areas in each part of Taiwan. From March 1, 2010, to April 30, 2013, 53 walk-in screenings were conducted among these 59 branches of the MPC. Each screening at each branch lasted 1 day to provide medical and charitable services to the general population as well as screening for dementia using AD8 [25], depression using the Chinese version of the CES-D (Center for Epidemiologic Studies-Depression) scale [26, 27], and sleep disorders using the Pittsburgh Sleep Quality Index PSQI [28]. For all 53 screenings of the general population, 7 screenings were conducted in the northern part, 19 in the central part, 24 in the southern part, and 3 in the eastern parts of Taiwan.

### 2.3. Center for Epidemiological Studies Depression Scale

The CES-D was used to measure the levels of depressive symptoms among adults. The CES-D consists of 16 negative affect and 4 positive affect items, such as “I felt depressed,” “I felt lonely,” and “I was happy.” Participants were asked regarding the number of days they experienced depressive symptoms during the previous week. Each item was accompanied by a standard 4-point Likert-scale of potential responses: 1: none, 2: 1 or 2 days a week, 3: 3 or 4 days per week, and 4: 5 days or more per week. Persons scoring more than 16 points on the CES-D scale were considered as having depression [3]. In the scale, 4 items that described positive affect were reversed before conducting our analysis. The Chinese version of this scale has been validated [29] and extensively used in studies of Chinese adults.

### 2.4. Participants and Evaluation

All participants were volunteers who joined the screening activity without any reward. The CES-D, AD8, and PSQI were administered to people after identifying their age, sex, and residence location. The participant was suspected of depression if his/her CES-D total score was greater than 16. All procedures were approved by the Kaohsiung Medical University Hospital Institutional Review Board (IRB). All information related to privacy or that could be identified was not recorded during the screening process.

### 2.5. Statistics

Data analysis was performed using SPSS (version 12.0.1 for Windows, SPSS Inc., Chicago, IL, USA). All statistical tests were 2-tailed, and an alpha of 0.05 was taken to indicate significance. Analysis of variance (ANOVA) was used to compare the difference of group mean for age and for CES-D total score among the 4 areas of Taiwan for all participants and for all suspected depression persons. The chi-square test was used to compare the proportion of suspected depression persons and sex between the 4 areas of Northern, Central, Southern, and Eastern Taiwan and among suspected depression participants.

## 3. Results

In total, 1612 participants, 131 in Northern, 494 in Central, 718 in Southern, and 269 in Eastern Taiwan were recruited with a mean age of 62.9 ± 14.4 years. The mean age was significantly different among the 4 areas (*P* < .001). The age of participants in Eastern Taiwan was older (mean ± SD: 69.8 ± 11.9) than that in the other 3 areas (Table 1). Participants were predominantly female; however, no significant difference existed in sex proportion (*P* = .634), although it was higher in the northern area (72.5%), compared with the other areas (Table 1). Among the total population, the mean ± SD of the CES-D score was 7.1 ± 8.7, with a significant difference between the 4 areas (*P* = .012). The highest CES-D score was in the southern area (mean ± SD: 7.8 ± 8.4), and the lowest score was in the eastern area (mean ± SD: 5.8 ± 9.4) (Table 1).

Among the ≥65-year-old participants, 772 participants, including 67 in Northern, 166 in Central, 359 in Southern, and 180 in Eastern Taiwan were recruited with a mean ± SD age of 74.8 ± 6.6 years. The mean age significantly differed among the 4 areas (*P* < .001). The age of participants in Eastern Taiwan was higher (mean ± SD: 76.5 ± 6.7) than in the other 3 areas (Table 2). Participants were predominantly female, but no significant difference existed in sex proportion (*P* = .953), although it was higher in the southern area (67.5%) compared with the other areas (Table 2). Among the ≥65-year-old participants, the mean of the CES-D score was 6.0 ± 8.2, with significant differences among the 4 areas. The highest CES-D score was in the southern area (mean ± SD: 7.2 ± 8.0), and the lowest score was in the central area (mean ± SD: 4.5 ± 6.7) (Table 2).

Among the total participants, the ratio of suspected depression participants did not significantly differ among the 4 areas (*P* = .675), nor was there a significant difference with respect to sex (*P* = .154) or the mean CES-D score (*P* = .067) (Table 3). The ratio of suspected depression patients was 16.4% of all recruited participants, with a mean CES-D score of 23.3 ± 7.0, mean age of 58.4 ± 16.8 years, and predominantly female 66.8% (Table 3). The mean age among the 4 areas significantly differed (*P* < .001) among the total population with respect to suspected depression. The participants were older in the eastern area (mean ± SD: 67.8 ± 14.7) and younger in the central area (mean ± SD: 51.4 ± 15.2) (Table 3).

Among the ≥65-year-old participants, the ratio of suspected depression participants was nonsignificantly different among the 4 areas (*P* = .128) as well as age (*P* = .315) and the mean CES-D score (*P* = .141) (Table 4). Among the ≥65-year-old participants, the ratio of suspected depression patients was 13.3% of all aged participants, with a mean CES-D score of 23.0 ± 7.4, mean age of 75.0 ± 7.1 years, and predominantly female 65.1% (Table 4). The sex ratio among the 4 areas significantly differed (*P* = .048) among the ≥65-year-old population with suspected depression. The ratio was highest in the eastern area (73.1%) and lowest in the central area (33.3%).

## 4. Discussion

This study provides updated information of the current status of depression among the general Taiwanese population and compares the difference among the 4 areas in Taiwan. Most of our recruited participants were female, both in the total population and in the ≥65-year-old population. Such results might partially indicate that females are more inclined to participate in social facilities than males, particularly for those featured with religion. Among the total or aged population, the CES-D score was significantly higher in the southern area. Despite our new findings of the higher proportion of depression in Southern Taiwan, no updated related studies address this issue. Further studies using randomized sampling are necessary to clarify such issues.

We also observed that the mean ages of all recruited and aged populations were significantly older in the eastern area, compared to the other areas in Taiwan. Such findings resulted from the status that the prevalence of the aged population in the eastern area is actually higher than the other areas, according to the recent “Report on the Survey of Citizens' Life Status in the Taiwan Area” published by the government, which indicates that 11.53% of the population in Taiwan is aged, with 10.76% in the northern area, 11.79% in the central area, 12.39% in the southern area, and 13.41% in the eastern area, as of 31 December, 2013 [30].

These suspected depressed patients, CES-D ≥16, were also predominantly female, which is similar to previous studies that found gender differences in depression prevalence, with women experiencing major depression approximately twice as often as men [31–34]. Several risk factors have been studied, which might account for gender differences in depression prevalence, including gender differences in hormones, socialization, coping style to stressful life events, and cultural influences [35, 36].

The mean age of these suspected depression participants was also significantly higher in the eastern area, which may result from the higher prevalence of the aged population in this area. Among the total population, the prevalence of suspected depression was 16.4%, which was higher than the prevalence screening of 8.9% among the more 15-year-old population in 2002 [37]. Certain studies have announced the rising incidence of depression since the early 20th century [31]. Several reasons may account for this, such as various study designs and tools, although most studies have indicated substantial socioeconomic changes.

Among the aged population, the prevalence of suspected depression was 13.3%. A previous study [38] showed 15.3% depressive neurosis and 5.9% major depression in the older adult community; however, other studies have found the prevalence of geriatric depression to be approximately 20.0% [39]. A comparison of these studies is difficult because of differences in research methodology, study population, diagnostic criteria, and instruments used [38]. These suspected depressed patients among the aged population were also predominantly female, except in the central area, which recruited fewer female participants in all recruited and suspected depressed participants among the 4 areas.

The results of this study could also provide information regarding the prevalence of mood disorder, anxiety, and depression in Taiwan, because depression and anxiety disorders are highly interrelated and frequently overlap [40].

This study has several strengths. First, we used the same interviewers to administer the CES-D instrument in all screenings to reduce interrater differences for the study results. Secondly, we used the same instrument, CES-D, to avoid biases from various assessment tools used in different screening sites. Third, we recruited participants from Northern, Central, Southern, and Eastern Taiwan to reflect the overall status of depression in Taiwan, which would be more objective, compared with other published studies limited to a certain area [15, 16, 18, 19]. However, this study has several limitations. First, we used a nonrandom sample of the population, such that the results cannot reflect the prevalence and incidence of current depression in Taiwan. Secondly, we used the CES-D alone, without other information to provide a clinical diagnosis of each suspected depression patient. However, the CES-D has been validated with reliable sensitivity and specificity in screening depression [24], and this study was focused on screening, not diagnosing, depression. Third, this study used a “walk-in” screening, which means that only persons who could partake in our screening program were recruited. A person who was dependent or could not be included in our screening sites was not allowed to join the screening program, so that our results would underestimate the actual status of depression prevalence.

This was a screening study with certain limitations and strengths, but with extensive coverage of Taiwan. We suggest a future study using randomized sampling to examine the prevalence and incidence of depression in Taiwan.

## Figures and Tables

**Table 1:** Demographic characteristics of all recruited participants.

*N* = 1612						
	Northern	Central	Southern	Eastern	*P* value	Total
Number (*n*, %)	131 8.1%	494 30.7%	718 44.5%	269 16.7%		1612 100%
Age, years (mean ± SD)	65.2 ± 12.3	58.2 ± 13.7	63.0 ± 14.8	69.8 ± 11.9	**<0.001**	62.9 ± 14.4
Sex (Female/*n*, %)	95 72.5%	320 67.1%	484 69.6%	186 69.1%	0.634	1085 69.0%
CES-D score (mean ± SD)	7.1 ± 9.7	6.8 ± 8.5	7.8 ± 8.4	5.8 ± 9.4	**0.012**	7.1 ± 8.7

**Table 2:** Demographic characteristics of recruited aged participants^#^.

*N* = 772						
	Northern	Central	Southern	Eastern	*P* value	Total
Number (*n*, %)	67 8.7%	166 21.6%	359 46.4%	180 23.3%		772 100%
Age, years (mean ± SD)	75.2 ± 6.7	73.1 ± 6.5	74.6 ± 6.4	76.5 ± 6.7	**<0.001**	74.8 ± 6.6
Sex (Female/*n*, %)	43 64.2%	106 66.3%	239 67.5%	119 66.1%	0.953	507 66.6%
CES-D score (mean ± SD)	5.0 ± 8.3	4.5 ± 6.7	7.2 ± 8.0	5.5 ± 9.3	**0.002**	6.0 ± 8.2

^#^Age ≥65 y/o participants.

**Table 3:** Demographic characteristic of participants suspected depression∗ among all recruited participants.

*N* = 265						
Recruited subjects	Northern (*N* = 131)	Central (*N* = 494)	Southern (*N* = 718)	Eastern (*N* = 269)	*P* value	Total (*N* = 1612)
Number (*n*, %)	21 16.0%	75 15.2%	127 17.7%	42 15.6%	0.675	265 16.4%
Age, years (mean ± SD)	56.4 ± 14.8	51.4 ± 15.2	59.7 ± 17.1	67.8 ± 14.7	**<0.001**	58.4 ± 16.8
Sex (Female/*n*, %)	17 81.0%	43 58.9%	87 70.7%	26 61.9%	0.154	173 66.8%
CES-D score (mean ± SD)	25.2 ± 10.6	23.3 ± 6.6	22.3 ± 6.0	25.1 ± 7.8	0.067	23.2 ± 7.0

*Defined as CES-D total score ≧16.

**Table 4:** Demographic characteristic of participants suspected depression∗ among aged participants^#^.

*N* = 103						
Recruited subjects	Northern (*N* = 67)	Central (*N* = 166)	Southern (*N* = 359)	Eastern (*N* = 180)	*P* value	Total (*N* = 772)
Number (*n*, %)	6 9.0%	15 9.0%	56 15.6%	26 14.4%	0.128	103 13.3%
Age, years (mean ± SD)	74.3 ± 8.9	73.4 ± 7.0	74.5 ± 7.0	77.2 ± 6.8	0.315	75.0 ± 7.1
Sex (Female/*n*, %)	4 66.7%	5 33.3%	39 69.6%	19 73.1%	**0.048**	67 65.1%
CES-D score (mean ± SD)	25.8 ± 13.9	21.7 ± 5.6	21.9 ± 6.2	25.5 ± 8.3	0.141	23.0 ± 7.4

*Defined as CES-D total score ≧16; ^#^Age ≥65 y/o participants.
